# 

**Published:** 1902-11

**Authors:** 


### BOOKS RECEIVED.

Manual of Gynecology by Henry T. Byford, M. D , Professor of Gynecology and Clinical Gynecology in the College of Physicians and Surgeons, Chicago; Professor of Gynecology in the Post-Graduate Medical School and in the Chicago Clinical School. Third revised edition. Duodecimo, pp. 620. Illustrated. Philadelphia: P. Blakiston's Son & Co. 1902. [Price, $3.00 net.]

Transactions of the State Medical Society of Wisconsin. Fifty-sixth annual meeting held at Milwaukee, June 4–6, 1902. Charles S. Sheldon, M. D., Secretary.

A Textbook of Anatomy. By American Authors. Edited by Frederic Henry Gerrish, M. D., Professor of Anatomy in the Medical School of Maine, Bowdoin College. Second Edition, thoroughly revised and enlarged. In one imperial octavo volume of 943 pages, with 1003 engravings in black and colors. Philadelphia and New York: Lea Brothers & Co., Publishers. 1902. [Cloth, $6.50 net; leather, $7.50 net; flexible water-proof binding, for use on the dissecting table, $7.00 net.]

A Manual of Surgery. For Students and Practitioners.By William Rose, M.B., B. S., Lond., F. R. C. S.; Professor of Clinical Surgery in King's College, London, and Senior Surgeon to King's College Hospital, and Albert Carless, M. S., Lond., F. R. C. S., Surgeon to King's College Hospital and Teacher of Operative Surgery in King's College, London; Examiner in Surgery to Glasgow University. Fifth edition. Octavo, pp. 1227. Illustrated. New York: William Wood & Company. 1902. [Price, $5.00 net.]

A Guide to the Practical Examination of Urine. For the use of Students and Physicians. By James Tyson, M. D., Professor of Medicine in the University of Pennsylvania, and Physician to the Hospital of the University. Tenth edition, revised and corrected. Philadelphia: P. Blakiston's Son & Co. 1902. [Price, $1.50.]

Transactions of the Medical Association of the State of Alabama. Annual meeting held at Birmingham, April 15–18, 1902. G. P. Waller, M. D., Secretary.

The Medical Directory of New York, New Jersey and Connecticut. Published by the New York State Medical, 64 Madison Avenue, New York. Volume IV. 1902–1903.

Blakiston's Quiz Compends. A Compend of Human Physiology. Especially adapted for the use of Medical Students. By Albert P. Brubaker, A. M., M. D., Adjunct Professor of Physiology and Hygiene in the Jefferson Medical College. Eleventh edition, revised and enlarged. Illustrated. Philadelphia: P. Blakiston's Son & Co. 1902. [Price, 80 cents.]

The Medical Epitomé Series. Genitourinary and Venereal Diseases. A Manual for Students and Practitioners. By Louis E. Schmidt, M. Sc., M. D., Associate Professor of Genitourinary Diseases, Chicago Polyclinic. Series edited by V. C. Pedersen, A. M., M. D.. Recently assistant demonstrator of Anatomy, College of Physicians and Surgeons, Columbia University, New York. Duodecimo, pp. 249. Illustrated with 21 engravings. Lea Brothers & Co. Philadelphia and New York. 1902.

